# Supplementary material for: Impact of COVID-19 on mucormycosis presentation and laboratory values: A comparative analysis
Source: PLoS One. 2025 May 2;20(5):e0321897. doi: 10.1371/journal.pone.0321897 (PMC12047787; doi:10.1371/journal.pone.0321897)
Supplement: S2 File — (DOCX) [file pone.0321897.s002.docx]

**Supplementary 2**

Table 8. Summary of GEE Results (Sex and Age as covariates)

| **Variable** | **P-value (Age)** | **Significance (Age)** | **Association Direction (Age)** | **P-value (Sex1)** | **Significance (Sex1)** | **Association Direction (Sex1)** |
| --- | --- | --- | --- | --- | --- | --- |
| WBC | 0.866 | NS | - | 0.018 | * | + |
| PMN | 0.608 | NS | - | 0.033 | * | - |
| Lym | 0.021 | * | + | 0.301 | NS | + |
| HB | 0.533 | NS | + | 0.155 | NS | + |
| PLT | 0.798 | NS | + | 0.154 | NS | - |
| AST | 0.19506 | NS | + | 0.5293 | NS | + |
| ALT | 0.078 | . | + | 0.618 | NS | + |
| ALP | 0.756 | NS | - | 0.641 | NS | - |
| ESR | 0.9184 | NS | - | 0.4231 | NS | + |
| CRP | 0.00053 | ** | + | < 2e-16 | ** | + |
| LDH | 0.3677 | NS | - | 0.1036 | NS | - |
| Facial Parenthesis | 0.9215 | NS | + | 0.00053 | ** | + |
| Facial Swelling | 0.866 | NS | - | 0.018 | * | + |
| Periorbital Swelling | 0.608 | NS | - | 0.033 | * | - |
| Sinus Pain | 0.021 | * | + | 0.301 | NS | + |
| Proptosis | 0.533 | NS | + | 0.155 | NS | + |
| Ptosis | 0.798 | NS | + | 0.154 | NS | - |
| Ophthalmic Pain | 0.19506 | NS | + | 0.5293 | NS | + |
| Ophthalmoplegia | 0.078 | . | + | 0.618 | NS | + |
| Blurred Vision | 0.756 | NS | - | 0.641 | NS | - |
| Otalgia | 0.9184 | NS | - | 0.4231 | NS | + |
| Nasal Discharge | 0.00053 | ** | + | < 2e-16 | ** | + |
| Headache | 0.3677 | NS | - | 0.1036 | NS | - |
| Dyspnea | 0.9215 | NS | + | 0.00053 | ** | + |
| Fever | 0.866 | NS | - | 0.018 | * | + |
| LOC | 0.608 | NS | - | 0.033 | * | - |
| Mortality | 0.021 | * | + | 0.301 | NS | + |
