# Supplementary material for: Impact of COVID-19 on mucormycosis presentation and laboratory values: A comparative analysis
Source: PLoS One. 2025 May 2;20(5):e0321897. doi: 10.1371/journal.pone.0321897 (PMC12047787; doi:10.1371/journal.pone.0321897)
Supplement: Attachment — Submitted filename: answer_to_reviewers (2).docx [file pone.0321897.s004.docx]

**Reviewer #1**

**Comment #1**: The authors should describe what criteria they used to define mucormycosis with respect to culture and pathology, citing any already defined literature.

**Response #1**: We thank the reviewer for this important point. To clarify, our study relied solely on histopathological examination for the diagnosis of mucormycosis. The exact criteria for mucormycosis detection in histopathological examination was added with a citation to a recent paper about mucormycosis histopathology in COVID-19 patients. *Please kindly refer to Inclusion Criteria Section.*

**Comment #2**: The frequency of mucomycosis was 102 cases per year at the two institutions more than past reports (10.1111/myc.12474). The appropriateness of the frequency in light of Iranian epidemiological data needs to be discussed along with the method of definition.

**Response #2**: We appreciate the reviewer's comment regarding the epidemiological context of mucormycosis in Iran. To address this, we have added the following paragraph to the Discussion section:

- " *The remarkably high frequency of mucormycosis cases in our study—102 within a single year—is significantly greater than previously reported figures in Iran. For instance, a systematic review spanning 25 years (1990-2015) documented only 98 cases nationwide. in Khuzestan province, 20 biopsy-proven cases were reported over a decade from 2004 to 2014 (43), highlighting the rarity of mucormycosis during that period. Evidence of a rising trend existed even before the COVID-19 pandemic. A retrospective study covering 208 cases from 2008 to 2014 showed a rising trend, with cases increasing from 9.7% in 2008 to 23.7% in 2014 (44, 45). The sharp increase in our study aligns with recent findings during the COVID-19 pandemic. Fazeli et al. (2021) reported a significant surge in rhino-orbital mucormycosis cases among COVID-19 patients in Kermanshah, with risk factors including diabetes and immunosuppressive therapy such as corticosteroid use (46). Additionally, Darazam et al. (2023) reported a 4.6-fold rise in cases during COVID-19 pandemic (47). Our findings mirror this trend, underscoring the significant impact of the COVID-19 pandemic on the epidemiology of mucormycosis in Iran.”*

**Comment #3**: Similarly, the authors should describe in detail the symptoms defined as COVID-19.

**Response #3:** Based on your valuable feedback, we have incorporated a detailed list of clinical symptoms used to screen for COVID-19, which was derived from established literature (reference 16), including fever, cough with sputum production, smell and taste disturbances, fatigue, and shortness of breath. Additionally, we provided clear criteria for the control group selection.

- Added section: *“Clinical symptoms suggestive of COVID-19 included fever, cough with sputum production, smell and taste disturbances, fatigue, and shortness of breath (16).”*

**Comment #4:** It should also be noted which manufacturer's equipment was used for PCR testing.

**Response #4:** Thank you for your feedback. The manufacturing details are added in the following format:

- “*For the molecular detection of COVID-19, the laboratory protocol employed the Rotor-Gene Q (Qiagen, Germany) PCR system coupled with the Magcore ® (RBC Bioscience Corp., Taiwan) extraction device to process and analyze patient* samples.”

**Comment #5:** The authors should also describe how long after the onset of COVID-19 disease mucormycosis are included in the positive group; the positive group is considerably more numerous than the control group.

**Response #5:** We appreciate your insightful feedback regarding the temporal relationship between COVID-19 infection and subsequent mucormycosis development. In our retrospective cohort study, we utilized electronic health records to identify patients who developed mucormycosis following their COVID-19 infections. Through PCR testing, we confirmed that COVID-19 infection preceded the mucormycosis diagnosis in all cases. However, our dataset had limitations in determining the exact interval between initial COVID-19 onset and the subsequent development of mucormycosis. While we can definitively state that COVID-19 occurred first in every case, we were unable to establish precise time intervals between the two conditions for inclusion criteria in our COVID-19 positive group.

**Comment #6:** It would be desirable to compare the percentage of COVID-19-positive patients who developed fungal infections with the previous literature.

**Response #6:** Thanks for your comment. We would like to highlight that due to the cross-sectional nature of our study and limitations in our dataset, we were unable to assess the percentage of COVID-19 patients who developed mucormycosis. Our study focused on patients who were diagnosed with mucormycosis, and we collected data on their COVID-19 status at the time of mucormycosis diagnosis. However, we did not have access to the total number of COVID-19-positive patients in the broader population from which our mucormycosis cases were drawn. This means we could not calculate the incidence or proportion of mucormycosis among all COVID-19 patients.

**Comment #7:** Authors should provide a breakdown of mycosis by species (candida, aspergills, etc.) and disease name for each group.

**Response #7:** We once again thank for your feedback. Our study relied solely on histopathological examination for the diagnosis of mucormycosis therefore we don’t have details regarding breakdown of mycosis by species.

**Comment #8:** The method section does not describe the extraction method, definition, etc. for initial clinical complaints. The authors should describe it in detail.

**Response #8:** We have expanded the methods section to provide detailed information about our COVID-19 case definition and extraction methodology. Specifically, we added comprehensive criteria for identifying the exposure group, which included both current and historical COVID-19 infections. The definition encompasses both laboratory confirmation (positive PCR tests) and radiological evidence.

- Added paragraph: *“Patients were included in the exposure group if they had either a current or historical COVID-19 infection. A current COVID-19 infection was defined by a positive COVID-19 PCR test or a chest X-ray highly suggestive of COVID-19. Additionally, individuals with a history of a positive COVID-19 PCR test at any point during the COVID-19 pandemic or chest X-ray findings indicative of a previous COVID-19 infection were also included in the exposure group. Chest X-ray findings suggestive of COVID-19 included primary features of atypical or organizing pneumonia, characterized by patchy or diffuse airspace opacities, whether consolidation or ground-glass opacity (14, 15). Patients without a positive COVID-19 PCR test and absence of these clinical symptoms were entered into the control group.”*

Also, we acknowledge the inherent limitations in retrospective chart review studies, particularly regarding the standardization of symptom documentation. In our study, clinical symptoms were extracted from hospitalization records that were completed by medical students and residents during routine clinical care. We recognize that without prospectively established criteria, there may be variability in how different clinicians defined and documented these symptoms.

- We have explicitly acknowledged this as a study limitation in our manuscript.
  - *“Several limitations warrant consideration in interpreting our findings. The study's retrospective design and reliance on self-reported history to determine prior COVID-19 infection status in the control group introduced potential selection bias, as we lacked definitive laboratory confirmation. A notable limitation was our dependence on medical records completed by medical students and residents during routine clinical care, without standardized criteria for symptom definition and documentation. This variability in symptom reporting may have affected data consistency and completeness. The inherent limitations of COVID-19 PCR testing, including both false positive and false negative results, could have led to patient misclassification, potentially impacting the accuracy of our group assignments and subsequent analysis of COVID-19's relationship with mucormycosis. Future prospective studies with systematic testing protocols and standardized symptom assessment criteria would help address these methodological constraints.”*

**Comment #9:** It is not clear whether COVID-19 severity and length of hospitalization were included in the analysis without presentation of results. The authors need to clearly present or explain this point.

**Response #9:** Thank you for your feedback. We agree that factors such as COVID-19 severity and hospitalization duration are crucial in understanding patient outcomes. Unfortunately, our dataset did not include detailed information on the severity of COVID-19 infection or the length of hospitalization for COVID-19. These variables were mistakenly included in Table 1 of the Methods section. We have corrected this error by removing these variables from the table and have revised the Methods section to accurately reflect the variables included in our analysis.

**Comment #10:** The presence or absence of SARS-CoV2 vaccination and history of treatment for COVID-19 in eligible patients should also be presented.

**Response #10**: We thank the respected reviewer for the valuable comment. Regarding vaccination status, due to our data anonymization protocol, patient identifiers (including national IDs) were removed from the dataset to protect patient privacy. Without these identifiers, we cannot retrospectively access vaccination records. Furthermore, there is no centralized vaccination database in Iran that would allow us to verify this information without patient identifiers. Also, concerning previous COVID-19 treatment history, Iran does not currently maintain a centralized electronic health record system, and patient treatment records are maintained separately by individual hospitals. Without patient identifiers, we cannot track treatments received at other healthcare facilities. The initial data collection did not include this historical information, and we cannot retroactively obtain it while maintaining patient anonymity.

**Comment #11:** Table 2 shows the results of the extraction from the electronic records and should be shown in the results section, not the materials and methods section.

**Response #11**: Thank you for your insightful comment, the table was moved and corrected based on your feedback.

**Comment #12**: For every table, abbreviations should be annotated with their full names.
The number of cases in each category should be described (e.g., n=xx).

**Response #12:** We appreciate the attention to details; the corrections were made based on your comment.

**Reviewer #2**

**Comment #1:** The introduction offers a solid overview of the topic; however, it would be strengthened by a clearer statement of the research question and objectives, along with the inclusion of more recent references to emphasize the current state of research in this field.

**Response #1:** Thank you for your insightful feedback. We would like to mention that the introduction’s last paragraph was rewritten in the following manner, and multiple citations were added regarding the current state of research.

- *“This study aims to investigate the differences in progression and initial symptom presentation of mucormycosis among patients with and without COVID-19. Additionally, we will examine alterations in laboratory values in mucormycosis patients co-infected with COVID-19 compared to those without the COVID-19 virus. By addressing these objectives, we hope to contribute valuable insights into the clinical management of mucormycosis in the context of the COVID-19 virus, ultimately aiding in the development of more effective diagnostic and therapeutic approaches.”*

**Comment #2:** The methodology section may also address potential limitations, such as the retrospective nature of the study or the possibility of selection bias.

**Response #2:** Thank you for your valuable comment; The limitation section has been updated as you suggested.

- *“Several limitations warrant consideration in interpreting our findings. The study's retrospective design and reliance on self-reported history to determine prior COVID-19 infection status in the control group introduced potential selection bias, as we lacked definitive laboratory confirmation. A notable limitation was our dependence on medical records completed by medical students and residents during routine clinical care, without standardized criteria for symptom definition and documentation. This variability in symptom reporting may have affected data consistency and completeness. The inherent limitations of COVID-19 PCR testing, including both false positive and false negative results, could have led to patient misclassification, potentially impacting the accuracy of our group assignments and subsequent analysis of COVID-19's relationship with mucormycosis. Future prospective studies with systematic testing protocols and standardized symptom assessment criteria would help address these methodological constraints.”*

Also, we would like to clarify that the sample of 102 individuals in this study represents the entirety of the available data rather than a subset selected for specific characteristics. This approach minimizes selection bias, as no intentional filtering or sampling was applied based on age, sex, or other demographic factors.

Figure 1: Age Distribution by Sex: a) Boxplot Analysis, b)Density Plot

Based on the age and sex distribution visualized in the plots, we conclude that there is no substantial bias in terms of these demographic factors:

**Boxplot of Age by Sex:**

The boxplot shows that both sex groups have comparable age distributions, with similar ranges and median ages. Although the age range for Sex 1 is slightly broader, the median ages for both groups are close, and their interquartile ranges (IQR) overlap significantly.

This overlap in IQRs and the lack of substantial differences in central tendencies indicate that the age distribution is balanced across sex groups, with no signs of preferential selection for particular ages within either group.

**Density Plot of Age by Sex:**

The density plot reveals slight differences in the shapes of age distributions for the two sex groups. Sex 0 has a single peak around middle age, while Sex 1 displays a broader distribution with multiple subtle peaks, suggesting greater variation in age within this group.

Despite these shape differences, the curves show considerable overlap, indicating that both sex groups include individuals across a wide range of ages. This overlap supports the notion that both sexes are well-represented and that any observed differences are due to natural variation rather than selection bias.

In summary, these analyses confirm that the age and sex distributions in our sample are representative of the available data, without any notable selection bias. Both sexes exhibit diverse age ranges, allowing for a balanced and unbiased analysis of age and sex effects in this study. However, we have also acknowledged in the limitations section that a larger sample could further enhance the ability to detect smaller effect sizes and refine estimates for non-significant predictors.

**Comment #3:** There is no mention of how the sample size of 102 patients was determined. A lack of justification for the sample size can raise questions about the statistical power of the study.

Table 1: Effect Sizes and 95% Confidence Intervals for Predictors Across Continuous Outcomes

| Outcome | Predictor | Effect_Size | CI_Lower | CI_Upper |
| --- | --- | --- | --- | --- |
| Lab_WBC | (Intercept) | 11358.8 | 4199 | 22426.9 |
|  | Post_covid1 | 2292.2 | -1534 | 6271.7 |
|  | Age | -23.3 | -162 | 84.7 |
|  | Sex1 | -997.8 | -5736 | 2552.1 |
|  | PMH_Cancer1 | 315.9 | -7547 | 10712.1 |
|  | PMH_DM1 | 2774.7 | -1024 | 6297 |
|  | r.Immunosuppressive1 | -3160.9 | -8276 | 1169.9 |
| Lab_PMN | (Intercept) | 69.9728 | 57.764 | 80.953 |
|  | Post_covid1 | 0.6612 | -6.568 | 7.546 |
|  | Age | -0.0197 | -0.215 | 0.197 |
|  | Sex1 | 6.9255 | 1.065 | 13.22 |
|  | PMH_Cancer1 | -10.4603 | -26.365 | 1.831 |
|  | PMH_DM1 | 7.3731 | 2.065 | 12.996 |
|  | r.Immunosuppressive1 | 4.2642 | -2.803 | 11.865 |
| Lab_Lym | (Intercept) | 19.5751 | 11.66 | 28.2324 |
|  | Post_covid1 | 1.7979 | -2.55 | 6.4237 |
|  | Age | -0.0302 | -0.17 | 0.0927 |
|  | Sex1 | -4.2721 | -8.55 | -0.4127 |
|  | PMH_Cancer1 | 0.5709 | -6.78 | 9.1383 |
|  | PMH_DM1 | -4.5053 | -9.76 | 0.1476 |
|  | r.Immunosuppressive1 | -0.8947 | -5.46 | 3.8839 |
| Lab_Hb | (Intercept) | 10.4348 | 7.9751 | 13.4228 |
|  | Post_covid1 | 0.2842 | -0.8724 | 1.4284 |
|  | Age | 0.00181 | -0.0388 | 0.0455 |
|  | Sex1 | 1.75502 | 0.7661 | 2.7654 |
|  | PMH_Cancer1 | -3.40934 | -5.2267 | -1.6381 |
|  | PMH_DM1 | 0.51232 | -0.8264 | 1.9155 |
|  | r.Immunosuppressive1 | 0.45512 | -0.4856 | 1.3687 |
| Lab_Plt | (Intercept) | 234124 | 143740 | 342438 |
|  | Post_covid1 | 62315 | 2949 | 121706 |
|  | Age | -663 | -2099 | 695 |
|  | Sex1 | -34145 | -78529 | 8170 |
|  | PMH_Cancer1 | -136246 | -208284 | -57456 |
|  | PMH_DM1 | 20052 | -30918 | 76048 |
|  | r.Immunosuppressive1 | -15311 | -60234 | 26017 |
| Lab_AST | (Intercept) | 8.272 | -19.5321 | 34.66 |
|  | Post_covid1 | 0.252 | -13.643 | 14.25 |
|  | Age | 0.509 | 0.0809 | 0.98 |
|  | Sex1 | 7.53 | -7.6347 | 22.39 |
|  | PMH_Cancer1 | 12.012 | -18.7803 | 40.9 |
|  | PMH_DM1 | -8.271 | -27.8011 | 7.21 |
|  | r.Immunosuppressive1 | 4.27 | -11.6537 | 19.66 |
| Lab_ALT | (Intercept) | 34.1688 | 10.53 | 58.748 |
|  | Post_covid1 | 11.7138 | -3.59 | 27.5 |
|  | Age | -0.0142 | -0.43 | 0.377 |
|  | Sex1 | 5.6747 | -6.77 | 18.794 |
|  | PMH_Cancer1 | 4.4983 | -19.03 | 27.706 |
|  | PMH_DM1 | -7.1855 | -23.94 | 9.112 |
|  | r.Immunosuppressive1 | 5.3586 | -7.87 | 19.609 |
| Lab_ALP | (Intercept) | 159.5 | -66.12 | 328 |
|  | Post_covid1 | -2.3 | -112.44 | 94.8 |
|  | Age | 1.42 | -3.09 | 6.4 |
|  | Sex1 | 73 | -18.89 | 187.8 |
|  | PMH_Cancer1 | 89.38 | -60.64 | 312.6 |
|  | PMH_DM1 | 33.83 | -59.96 | 166.4 |
|  | r.Immunosuppressive1 | -42.79 | -155.65 | 43.9 |
| Lap_ESR | (Intercept) | 78.7172 | 48.22 | 105.925 |
|  | Post_covid1 | 2.5721 | -15.11 | 18.587 |
|  | Age | 0.0432 | -0.36 | 0.439 |
|  | Sex1 | -8.8645 | -21.52 | 4.273 |
|  | PMH_Cancer1 | 13.2105 | -11.37 | 36.664 |
|  | PMH_DM1 | -0.5912 | -15.86 | 14.296 |
|  | r.Immunosuppressive1 | -15.2084 | -28.72 | -2.403 |
| Lab_CRP | (Intercept) | 31.918 | -8.48 | 74.736 |
|  | Post_covid1 | 17.645 | -19.458 | 55.589 |
|  | Age | 0.405 | -0.222 | 1.053 |
|  | Sex1 | 6.33 | -13.916 | 27.241 |
|  | PMH_Cancer1 | 65.161 | 26.279 | 107.985 |
|  | PMH_DM1 | 34.685 | 14.446 | 55.652 |
|  | r.Immunosuppressive1 | -22.329 | -45.65 | 0.566 |
| Lab_LDH | (Intercept) | 626.21 | 327.23 | 964.64 |
|  | Post_covid1 | -29.14 | -193.37 | 143.66 |
|  | Age | 3.33 | -0.66 | 7.57 |
|  | Sex1 | 31.01 | -102.04 | 156.88 |
|  | PMH_Cancer1 | -2.8 | -259.39 | 292.76 |
|  | PMH_DM1 | -34.2 | -198.54 | 138.41 |
|  | r.Immunosuppressive1 | 127.96 | -13.22 | 265.16 |

**Response #3:** Thank you for your valuable feedback and for the opportunity to clarify the adequacy of our sample size. To begin, it is important to note that the sample size of 102 was not a result of specific sampling; rather, it represents the entirety of available data for this study. This ensures that the results are not influenced by sampling bias and reflect a comprehensive analysis of the population data accessible to us. Also, we offer the following evidence, based on statistical significance, effect sizes, and bootstrapped confidence intervals for our predictors across multiple continuous outcome variables:

**Significance and Robustness of Key Predictors:**

- Across the models, we observe that several predictors show consistent significance, indicating that the sample size provides adequate power to detect meaningful effects. For instance: Predictors with consistently low p-values include Post_covid1, PMH_Cancer1, and r.Immunosuppressive1 for multiple outcomes (e.g., Lab_PMN, Lab_Lym, Lab_Plt, and Lab_CRP). These predictors reach statistical significance with p-values well below 0.05 in multiple models, which demonstrates the adequacy of the sample size in capturing significant associations.

**Effect Sizes and Confidence Intervals:**

- To further illustrate the practical importance of significant predictors, we conducted a bootstrap analysis to calculate effect sizes and 95% confidence intervals for each predictor across all outcomes. For each significant predictor, the bootstrapped confidence intervals provide a range around the effect size, giving us additional insights into the robustness and magnitude of each effect. For example: Post_covid1 shows a positive effect size across outcomes like Lab_Plt (62,315, 95% CI: 2,949 to 121,706) and Lab_CRP (17.65, 95% CI: -19.46 to 55.59), indicating a likely association with higher values for these markers post-COVID. PMH_Cancer1 and PMH_DM1 also show meaningful effect sizes in outcomes such as Lab_CRP (PMH_Cancer1: 65.16, 95% CI: 26.28 to 107.99) and Lab_PMN (PMH_DM1: 7.37, 95% CI: 2.07 to 13.00), suggesting an increased level in inflammatory markers for patients with a history of cancer or diabetes. r.Immunosuppressive1 shows a negative effect on Lap_ESR (-15.21, 95% CI: -28.72 to -2.40), which may suggest a protective or reducing effect on ESR in patients on immunosuppressive therapy.

**Interpretation of Non-Significant Predictors:**

- Several predictors (e.g., Age, Sex) do not consistently show significant associations across outcomes. This suggests that the sample size is sufficient to distinguish between predictors with meaningful effects and those with weaker or no associations with the disease outcomes. While a larger sample size could potentially detect smaller effects in these variables, the current sample size was adequate to capture the primary associations of interest.

**Clinical Relevance of Effect Sizes:**

- While p-values indicate statistical significance, the effect sizes and confidence intervals provide clinical context. The substantial effect sizes for predictors like Post_covid1, PMH_Cancer1, and PMH_DM1 underscore that these associations are not only statistically significant but also potentially clinically meaningful.

In summary, the sample size of 102 is sufficient to detect robust associations in predictors with meaningful effects on the outcomes studied. The combination of significant p-values, effect sizes, and confidence intervals supports the adequacy of our sample size in capturing clinically relevant associations without being overly influenced by smaller, less meaningful effects. Thank you once again for allowing us to provide this detailed response. We believe this evidence collectively addresses the justification of our sample size and reinforces the robustness of our findings. However, we have also acknowledged in the limitations section that a larger sample could further enhance the ability to detect smaller effect sizes and refine estimates for non-significant predictors.

**Comment #4:** There may be a need for a more nuanced interpretation of the results, particularly regarding the associations found between COVID-19 history and clinical outcomes. The implications of these findings should be discussed in the context of existing literature.

**Response #4:** Thank you for your valuable feedback. Based on your feedback, we have provided more interpretation and explanations. *Please kindly refer to Discussion Section*.

**Comment #5:** The analysis should consider potential confounding variables that could influence the outcomes, such as the severity of COVID-19 or other underlying health conditions.

**Response #5**: Thanks for your insightful comment. We recognize that potential confounding factors like COVID-19 severity and underlying health conditions can significantly impact patient outcomes. While we were unable to include COVID-19 severity in our analysis due to data limitations, we did account for some underlying health conditions available in our dataset, such as diabetes and immunosuppression status.
