# Supplementary material for: Impact of COVID-19 on mucormycosis presentation and laboratory values: A comparative analysis
Source: PLoS One. 2025 May 2;20(5):e0321897. doi: 10.1371/journal.pone.0321897 (PMC12047787; doi:10.1371/journal.pone.0321897)
Supplement: Attachment — Submitted filename: answer to reviewer 2.docx [file pone.0321897.s005.docx]

**Response to Reviewer Comments**

We thank both reviewers for their insightful comments, which have significantly improved the manuscript. We have carefully addressed each point and made revisions accordingly.

**Response to Reviewer #1**

Thank you for your thorough review and helpful feedback. As suggested, we have thoroughly **rewritten the Limitations section** to more comprehensively convey the limitations of our study. This includes explicitly stating that:

- We were unable to determine the precise interval between COVID-19 onset and the development of mucormycosis due to data limitations.
- We were unable to investigate COVID-19 severity as a variable, as this information was not available in our dataset.
- We were unable to investigate vaccination status and past COVID-19 treatment due to the constraints of data anonymization and lack of a centralized database.

**Response to Reviewer #2**

We appreciate your feedback and have made the following changes to address your concerns:

- **Statistical Analysis Section:** We have updated the statistical analysis section to clearly state our intent for using Generalized Estimating Equations (GEE) for modeling correlated data within our study. We have also included a reference to support the use of GEE in our analysis approach.
- **Introduction Closing Paragraph:** We have revised the closing paragraph of the introduction to better highlight the specific goals of our study in light of the existing body of literature, focusing on the distinctive contributions of our research.
- **Discussion Opening Paragraph:** We have updated the opening section of the discussion to rephrase the significance of our findings and to emphasize the novel contribution our results make to the existing understanding of COVID-19 associated mucormycosis.
- **Clinical Implications:** We have included discussion that more clearly and directly points out the clinical implications of our findings, particularly in regards to the specific laboratory findings we report and their diagnostic value in the context of COVID-19 associated mucormycosis. We have also included 3 references to support the clinical significance.
- **Conclusion Section:** We have updated the conclusion section to reflect the changes and convey the revised goals of our study.
