# Supplementary material for: Impact of COVID-19 on mucormycosis presentation and laboratory values: A comparative analysis
Source: PLoS One. 2025 May 2;20(5):e0321897. doi: 10.1371/journal.pone.0321897 (PMC12047787; doi:10.1371/journal.pone.0321897)
Supplement: Attachment — Submitted filename: answer to reviewers 3.docx [file pone.0321897.s006.docx]

Thank you for your insightful and constructive comments on our manuscript. We appreciate your thorough review and have revised the manuscript to address each of your points. Below is a point-by-point response outlining the changes we have made (changes are highlighted with green in the marked manuscript):

**Point 1: Facial Swelling and COVID-19 Link**

We acknowledge your concern regarding the seemingly counterintuitive notion of COVID-19 reducing facial swelling in mucormycosis patients. We agree that this aspect requires careful consideration. As you suggested, we have clarified in the discussion section that our explanations regarding facial swelling are not definitive and that misclassification bias cannot be ruled out due to the retrospective nature of our study and potential inconsistencies in symptom documentation. We have incorporated the following text into the discussion to explicitly address this point:

"[While our proposed explanations for these patterns are plausible, it's important to recognize that we could not definitively confirm the precise underlying mechanism of altered facial swelling. Furthermore, due to the retrospective nature of our study and reliance on patient records where facial swelling was not consistently documented as a standardized symptom, the possibility of misclassification cannot be excluded, particularly in our cohort]"

**Point 2: Comparison with Prior Studies (Thrombocytosis, WBC, Fever Absence)**

We appreciate your suggestion to expand the discussion section to compare our results with prior studies. We have now expanded this section to include comparisons with existing literature, particularly focusing on thrombocytosis, WBC count changes, and fever absence. We have incorporated four new references to support these expanded explanations.

**Point 3: Platelet Counts in COVID-19 Patients**

We have carefully considered your comment regarding the higher platelet counts observed in our COVID-19 associated mucormycosis (CAM) patients. We have clarified in the manuscript that while other studies may not have reported increased platelet counts in CAM patients specifically, our results indicate a significant increase in PLT in our cohort. We have reinforced our explanation for this finding, highlighting the role of systemic inflammation driven by both COVID-19 and mucormycosis. We have provided references to support the link between inflammation, elevated CRP and D-dimer in COVID-19, and increased platelet production. Furthermore, we have explicitly acknowledged that our finding regarding thrombocytosis in CAM patients may differ from studies that focus on thrombocytopenia in severe COVID-19 alone. The following text exemplifies this clarification in the discussion:

"[The phenomenon is likely driven by the systemic inflammation inherent in both COVID-19 and mucormycosis, where the inflammatory milieu, characterized by elevated CRP and D-dimer in COVID-19, exacerbates platelet production(41-43).Clinically, these findings suggest that while thrombocytopenia may be a marker of severe COVID-19(44, 45), higher or normal platelet counts, particularly in the context of elevated inflammatory markers like CRP and D-dimer(41, 42), in a patient with suspected mucormycosis and a history of COVID-19 should not rule out the diagnosis. Instead, clinicians should consider the broader context of the patient's medical history and other laboratory findings, such as inflammatory markers, and recognize thrombocytosis as a potential indicator of CAM in the post-COVID-19 setting.]"

**Point 4: Verbal Consent and Ethics Approval**

We have revised the manuscript to explicitly state that verbal consent was formally recorded and approved by the Ethics Committee. We have included the ethics code and detailed the process of verbal consent recording that was approved and implemented. The following text has been updated in the manuscript:

"[The study was approved with the ethics code IR.MUMS.MEDICAL.REC.1401.054. The Ethics Committee of Mashhad University of Medical Sciences waived the requirement for patients' written consent and specifically approved the use of verbal informed consent. Furthermore, the ethics committee approved the method for formally recording this verbal consent, and this approved procedure was implemented for all participants prior to their study involvement.]"

Thank you again for your time and valuable comments.

Sincerely,
